# Clinical factors associated with uterine rupture in type II angular pregnancy: a 10-year single-institution retrospective study

**DOI:** 10.3389/fmed.2025.1656273

**Published:** 2025-09-24

**Authors:** Bao-You Huang, Portia Cobbinah, Hao-Ran Hu, Ya-Shi Zhu, Mei-Qin Yang, Jian-Yi Ding, Xin-xin Xu, Hui-juan Zhou, Bo Yin, Ling-Fei Han

**Affiliations:** ^1^Department of Gynecology, Shanghai Key Laboratory of Maternal Fetal Medicine, Shanghai Institute of Maternal-Fetal Medicine and Gynecologic Oncology, Shanghai First Maternity and Infant Hospital, School of Medicine, Tongji University, Shanghai, China; ^2^Department of Gynecology, The First Affiliated Hospital of Wenzhou Medical University, Wenzhou, Zhejiang, China; ^3^Medical Humanities and Management, Wenzhou Medical University, Wenzhou, China

**Keywords:** angular pregnancy, salpingectomy, uterine rupture, diagnostic ultrasonography, eccentric intrauterine pregnancy

## Abstract

**Objective:**

To introduce the classification and focus on retrospectively investigating clinical factors associated with uterine rupture.

**Materials and methods:**

We retrospectively analyzed 222 cases of angular pregnancies from January 2010 and December 2021. The selected cases were classified into two types, type I (*n* = 19) and type II (*n* = 199). Additionally, type II cases were further subdivided into the ruptured group (*n* = 25) and the unruptured group (*n* = 174). Clinical data were collected, and univariate and multivariate analyses were performed to identify significant indicators.

**Results:**

The mean maternal age was 31.5 ± 5.8 years, with a mean BMI (body mass index) of 22.0 ± 3.2 kg/m^2^ in 199 type II patients. Spontaneous uterine rupture occurred in 25 (12.6%) patients, while 174 (87.4%) remained unruptured. Univariate analysis revealed that abdominal pain (*P* < 0.001), a history of previous ipsilateral salpingectomy (*P* = 0.002), vaginal bleeding (*P* = 0.005), and gestational age (GA) ≥ 7 weeks (*P* = 0.044) were significant factors of rupture in type II angular pregnancy. Multivariate analysis identified abdominal pain (OR = 10.410, 95% CI: 3.286–32.977, *P* < 0.000) and ipsilateral salpingectomy (OR = 3.270, 95% CI: 1.209–8.847, *P* = 0.020) as statistically significant independent risk factors. The ruptured group had clinically and statistically significant lower hemoglobin and higher transfusion rates.

**Conclusion:**

The classification system of angular pregnancy (AP) is a valuable tool that facilitates appropriate management and good prognostic outcomes. Type I angular pregnancy can be followed up till term. Type II angular pregnancy is a high-risk form, and clinicians must carefully assess and investigate other factors such as the history of ipsilateral salpingectomy and abdominal pain and high alert for uterine rupture.

## Introduction

Angular pregnancy is characterized as an eccentric intrauterine pregnancy (1–3). Kelly in the 19th century, defined it as an embryo implant located at a lateral angle of the uterine cavity, medial to the utero-tubal junction (4). Even though the first mention of angular pregnancy is over a century ago, there remains a paucity of literature on the subject. The prognosis reported in different literature is indeed quite divergent. Some studies have shown that patients with angular pregnancy can achieve live births during close-interval follow-up (5–7). However, uterine rupture, one of the major complications of angular pregnancy, which can lead to severe intra-abdominal hemorrhage and consequent maternal death, has been reported in several studies (8–11).

**FIGURE 1:**
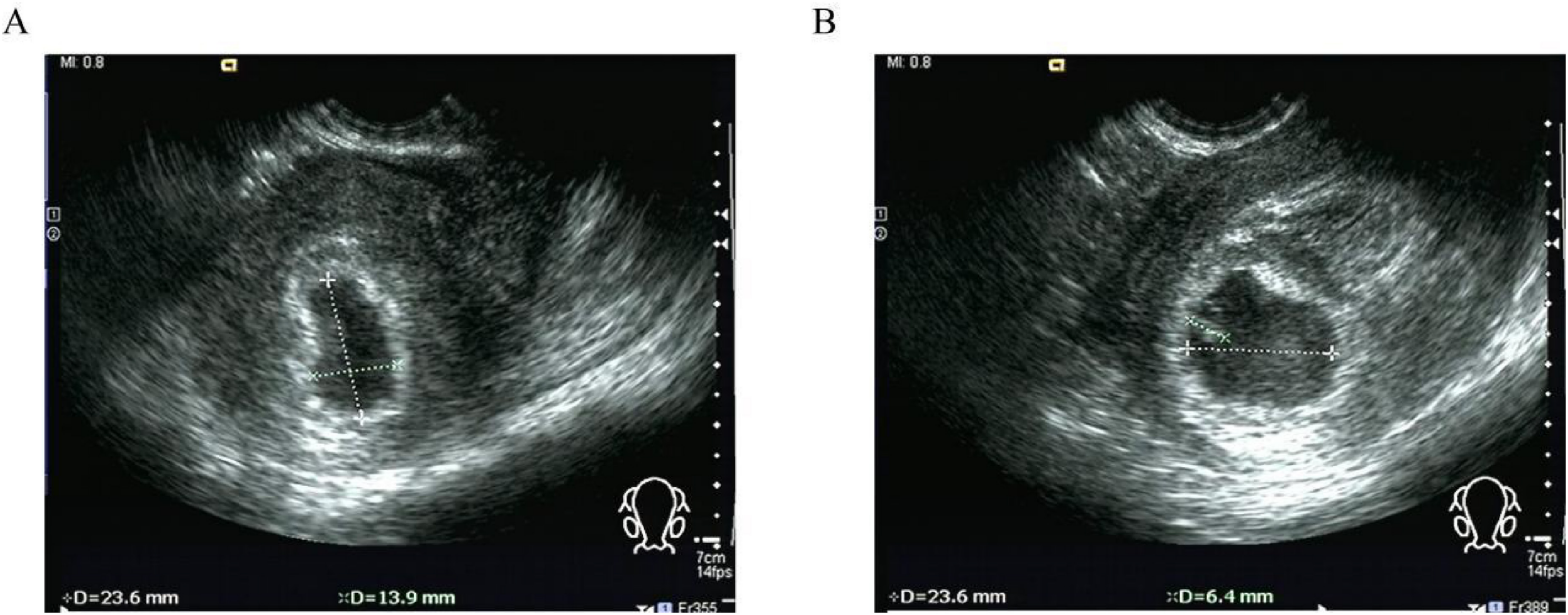
Type I angular pregnancy: **(A)** A gestational sac of about 24 mm × 14 mm × 24 mm in size was seen near the right uterine horn in the endometrium by transvaginal two-dimensional ultrasonography. **(B)** The echogenic embryo was seen inside the sac, with a long diameter of about 6 mm and a visible heart tube.

**FIGURE 2:**
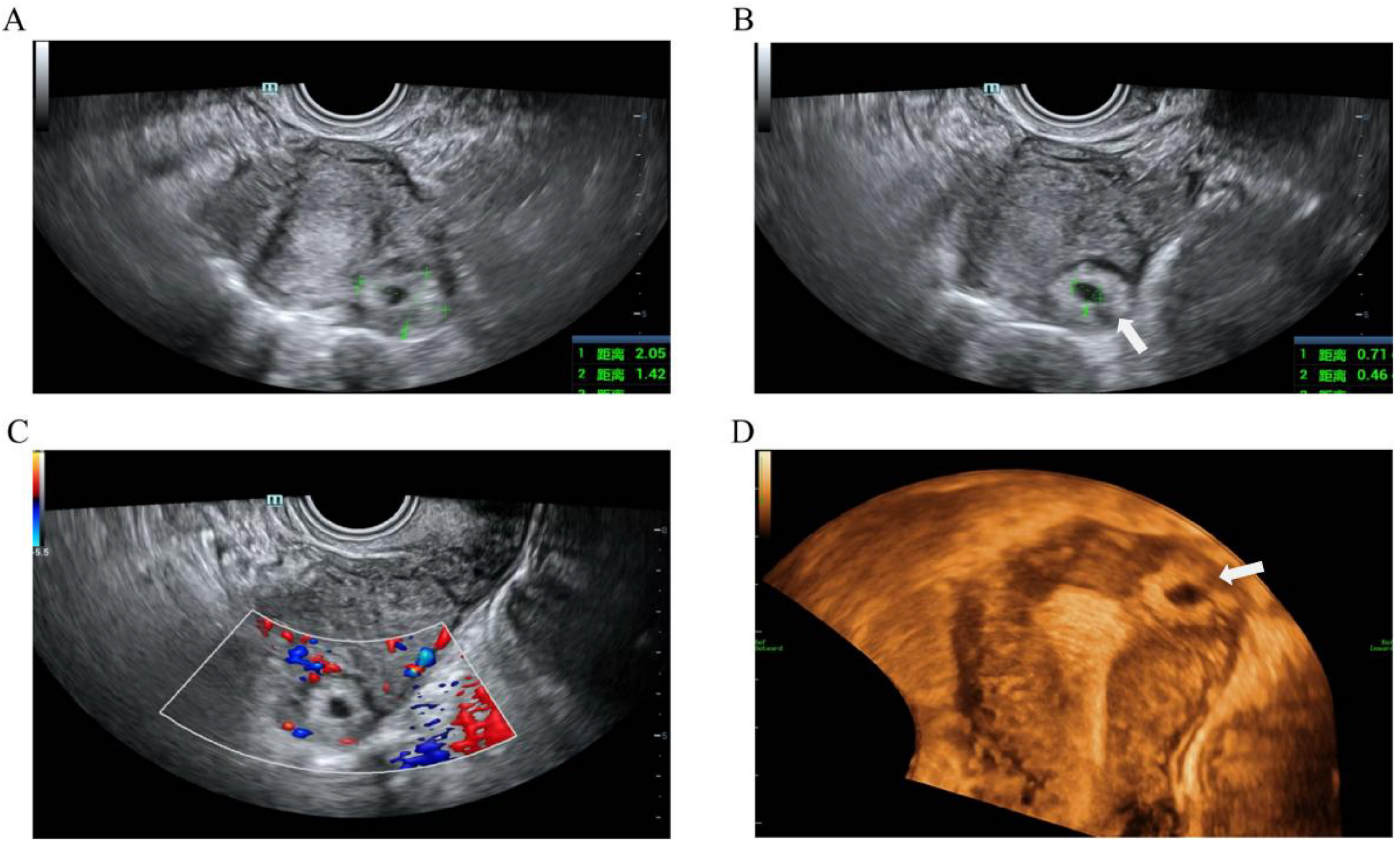
Type II angular pregnancy: **(A)** The two-dimensional ultrasound showed a mixed echo pattern mass measuring 21 mm × 14 mm × 18 mm in the left uterine angle, expanding toward the cornual region of the uterus. **(B)** A mixed echo identified a gestational sac measuring 7 mm × 5 mm × 6 mm. **(C)** Image of blood flow of AP in the left uterine angle. **(D)** The 3-dimensional ultrasound showed a mass adjacent to the endometrium, expanding toward the cornual region of the uterus. The arrow indicates a thin myometrial wall.

**FIGURE 3:**
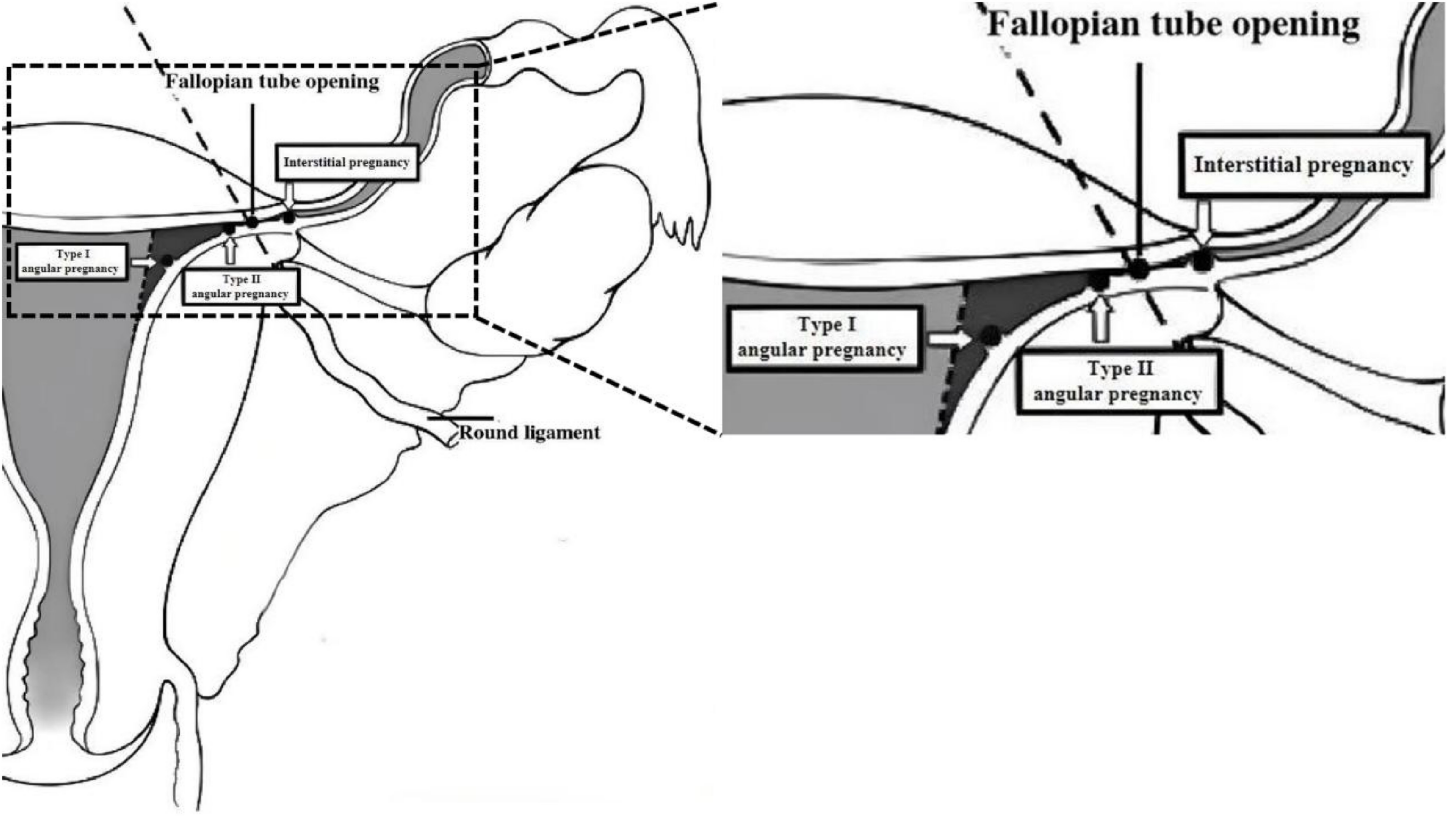
Angular pregnancy schematic diagram (12).

A classification system was proposed by Chinese experts in 2000 (12). Based on the gestational sac’s growth pattern, angular pregnancy is divided into type I and type II (12). The gestational sac of type I angular pregnancy is situated at the mediolateral junction of the uterine cavity, enveloped by two circular decidual bands and partially surrounded by a myometrial layer (Figure 1). The risk of uterine rupture is low, and the pregnancy usually progresses to the mid or late trimester. In contrast, type II angular pregnancy is characterized by the outward growth of the gestational sac toward the cornual region, resulting in significant bulging, associated with an elevated risk of uterine rupture and life-threatening hemorrhage (Figure 2). Utilizing this classification system enables clinicians to enhance their comprehension of diverse pathological processes, thus facilitating the selection of the most appropriate management options to ensure optimal patient prognoses. A diagnosis of angular pregnancy can be made by two-dimensional transvaginal/transabdominal ultrasound (Figure 2) and confirmed with a laparoscopy or laparotomy. The Figure 3 shows a schematic diagram of angular pregnancy.

In this study, we first introduce the classification of angular pregnancy to identify the type II patients requiring clinical intervention. Then we focus on retrospectively investigating clinical factors associated with uterine rupture in type II angular pregnancy. We hope our study will help clinicians develop appropriate treatment strategies and avoid adverse outcomes.

## Materials and methods

### Population and data collection

**FIGURE 4:**
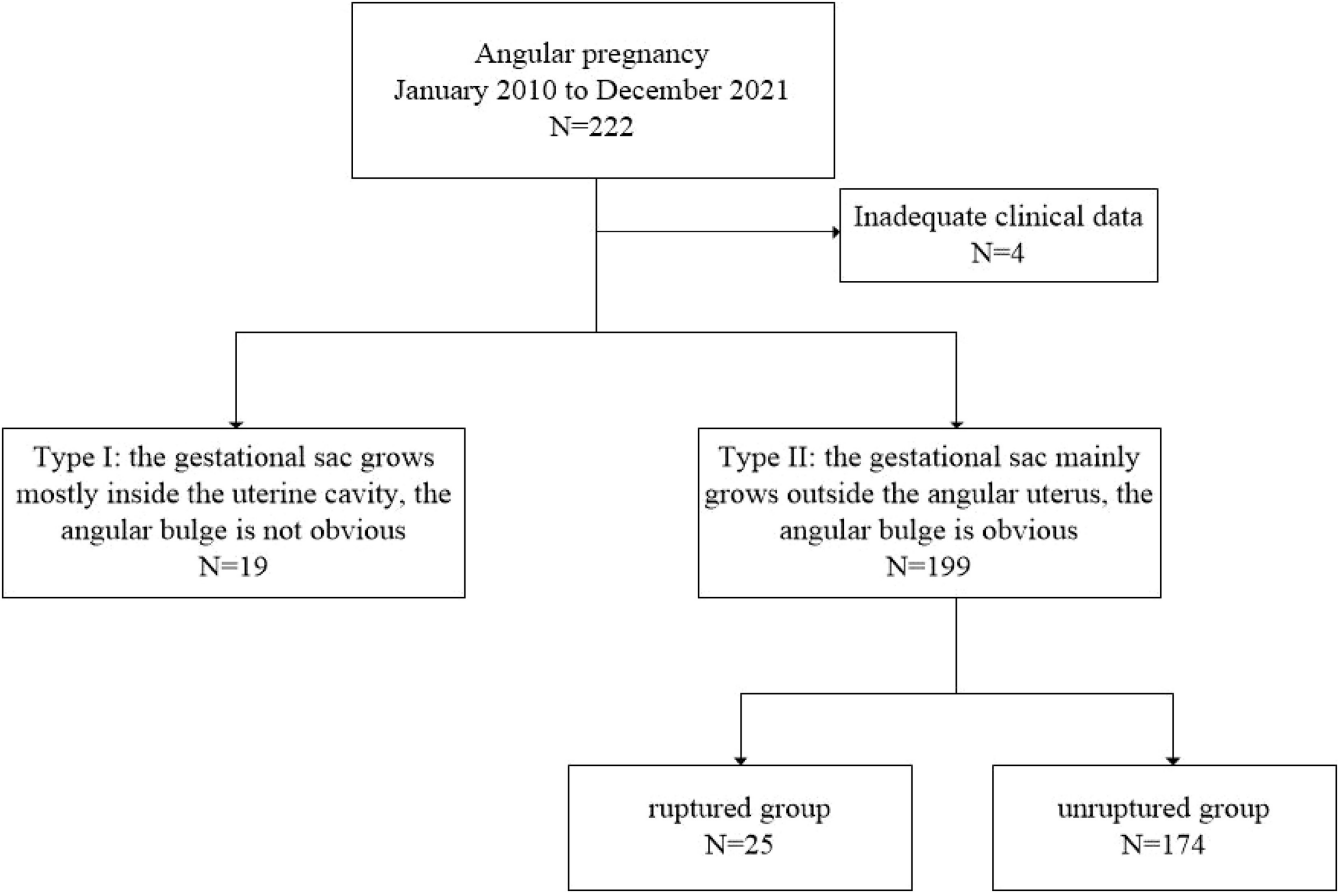
Flow chart of patient selection.

This study was approved by the Ethical Committee of the First Affiliated Hospital of Wenzhou Medical University with the number KY2022-R213, and written informed consent was obtained. A cohort of inpatients diagnosed with angular pregnancy who underwent surgery between January 2010 and December 2021 was collected. All patients were offered ultrasound scans during the first trimester of pregnancy. There were 222 patients with a confirmed diagnosis of angular pregnancy. Among them, 4 patients had incomplete data, 19 patients were diagnosed with type I, and 199 patients were diagnosed with type II. Additionally, according to the intraoperative status of the uterus, type II was further subdivided into ruptured group with 25 patients and unruptured group with 174 patients (Figure 4). The collected data included age (years), BMI (body mass index, calculated as weight in kilograms divided by height in meters squared), abortion history, gravidity, parity, IVF-ET (*in vitro* fertilization–embryo transfer), previous ectopic pregnancy, salpingectomy history, uterine fibroids or adenomyoma, and intraoperative uterine status. Postoperative histological examination confirmed chorionic villus tissue of the excised tissue in all patients.

### Statistical analysis

The SPSS software version 25 (IBM, Chicago, IL, USA) was used. For continuous variables, distributed data were presented as mean ± standard deviation (SD) and median (range) or median [interquartile range (IQR)]. Univariate and multivariate analyses were used to preliminarily screen out potentially significant indicators, and a 95% confidence interval (95% CI) of risk factors for the ruptured and unruptured groups, respectively. The difference in overall mean was explored using a two-sample Student’s *t*-test or Wilcoxon rank-sum test. Categorical variables were expressed as raw numbers or percentages using Fisher’s exact test or the Chi-square test. It is imperative to note that a two-sided test was used to carry out all statistical analyses. The statistical significance level was set at Q = 0.05, and *P* < 0.050 was considered statistically significant. Forest plot was drawn by GraphPad Prism 8.

## Results

### Clinical characteristics

The clinical characteristics of 199 type II angular pregnancies are shown in Table 1. The mean age was 31.5 ± 5.8 years, with a mean body mass index (BMI) of 22.0 ± 3.2 kg/m^2^. Previous salpingectomy surgery was documented in 70 (35.2%) patients. Of all these patients, 49 had abdominal pain, 54 had vaginal bleeding, and 66 had no symptoms. A mixed echogenic mass was detected in 39 (19.6%) patients, a gestational sac in 160 (80.4%) patients, and a heartbeat in 69 (34.7%) patients by ultrasound examination. The median preoperative serum HCG was 11,897 IU/L (838–22,500 IU/L). After surgery, uterine rupture was confirmed in 25 (12.6%) patients.

**TABLE 1:** Basic clinical characteristics of 199 type II angular pregnancies.

Characteristics	Patients (*N* = 199)
Age, years, mean ± SD	31.50 ± 5.665
BMI, kg/m^2^, mean ± SD	22.0659 ± 3.26091
Gravidity ≥ 1, *n* (%)	167 (83.9)
Parity ≥ 1, *n* (%)	125 (62.8)
Abortion ≥ 1, *n* (%)	140 (70.3)
IVF-ET, *n* (%)	34 (17.1)
Previous ectopic pregnancy, *n* (%)	46 (23.1)
Salpingectomy history, *n* (%)	70 (35.2)
**Symptoms, *n* (%)**	
Abdominal pain	49 (24.6)
Vaginal bleeding	54 (27.1)
Abdominal pain + vaginal bleeding	30 (15.1)
No symptoms	66 (33.2)
**Ultrasound, *n* (%)**	
Mixed echo mass	39 (19.6)
Gestational sac	160 (80.4)
Heartbeat	69 (34.7)
Uterine fibroids or adenomyoma, *n* (%)	37 (18.6)
Preoperative HCG, IU/L, median (range)	11,897 (838−22,500)
**Intraoperative status**	
Ruptured	25 (12.6)
Unruptured	174 (87.4)

BMI, body mass index; IVF-ET, *in vitro* fertilization and embryo transfer; HCG, human chorionic gonadotrophin.

Univariate analysis of clinical symptoms and specific clinical parameters between the two groups is shown in Table 2. The results showed that abdominal pain (*P* < 0.001), a history of previous ipsilateral salpingectomy (*P* = 0.002), vaginal bleeding (*P* = 0.005), and gestational age (GA) ≥ 7 weeks (*P* = 0.044) were significant factors. There were no statistically significant differences including age (*P* = 0.727), BMI (*P* = 0.236), abortion ≥ 1 (*P* = 0.093), gravidity ≥ 1 (*P* = 0.149), parity ≥ 1 (*P* = 0.896), IVF-ET (*P* = 1.000), history of ectopic pregnancy (*P* = 0.230), the presence of heartbeat (*P* = 0.099), combined with uterine myoma or adenomyoma (*P* = 0.388), and preoperative serum HCG levels (*P* = 0.601).

**TABLE 2:** Univariate analysis of factors related to rupture in type II angular pregnancy.

Parameter	Ruptured group (*N* = 25)	Unruptured group (*N* = 174)	*T*/χ^2^ value	*P*-value
Age, years, mean ± SD	31.1 ± 5.4	31.6 ± 5.8	−0.356	0.727
BMI, kg/m^2^, mean ± SD	21.3 ± 2.4	22.3 ± 3.3	−1.221	0.236
Abortion ≥ 1, *n* (%)	14 (56.0)	126 (72.4)	2.823	0.093
Gravidity ≥ 1, *n* (%)	18 (72.0)	149 (85.6)	3.010	0.149
Parity ≥ 1, *n* (%)	16 (64.0)	109 (62.6)	0.017	0.896
IVF-ET, *n* (%)	4 (16.0)	30 (17.2)	0.024	1.000
History of ectopic pregnancy, *n* (%)	8 (32.0)	37 (21.3)	1.270	0.230
Ipsilateral salpingectomy, *n* (%)	14 (56.0)	45 (25.7)	10.974	0.002*
Abdominal pain, *n* (%)	21 (84.0)	58 (33.3)	23.441	< 0.001*
Vaginal bleeding, *n* (%)	4 (16.0)	79 (45.4)	7.78	0.005*
Gestational age ≥ 7 w, *n* (%)	9 (36.0)	100 (57.5)	0.024	0.044*
Heartbeat, *n* (%)	5 (20.0)	64 (36.8)	3.063	0.099
Combined with uterine fibroids or adenomyoma, *n* (%)	7 (28.0)	32 (18.4)	1.672	0.388
Preoperative HCG, IU/L, median (IQR)	11,381 (3,349–31,314)	11,936.5 (4,502–32,068)	0.285	0.601

SD, standard deviation; BMI, body mass index; IVF-ET, *in vitro* fertilization and embryo transfer; HCG, human chorionic gonadotrophin.

*Represents *p* < 0.05.

**FIGURE 5:**
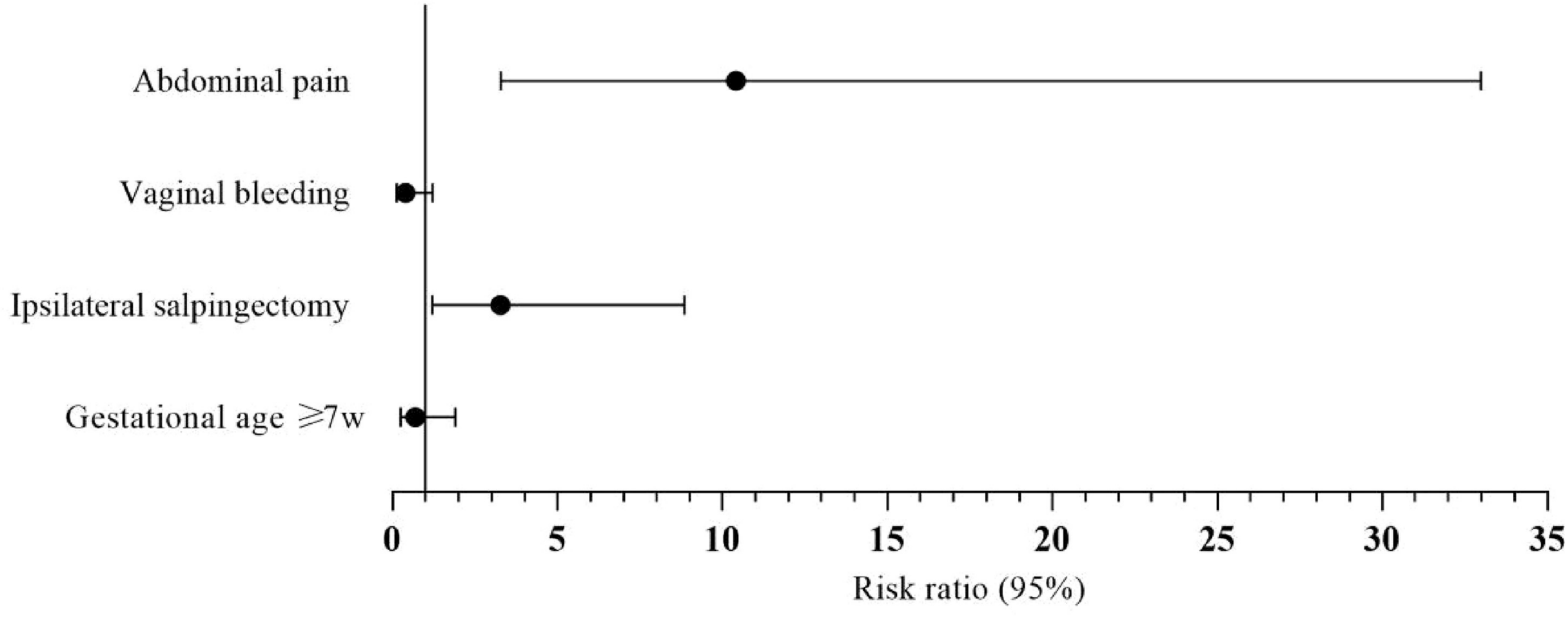
Forest plot showing the results of multivariate logistic regression analysis and visualizing the risk ratios of the characteristics for type II angular pregnancy.

All the related variables were entered into the multivariate logistic regression model. The analysis identified abdominal pain (OR = 10.410, 95% CI: 3.286–32.977, *P* < 0.000) and ipsilateral salpingectomy (OR = 3.270, 95% CI: 1.209–8.847, *P* = 0.020) as statistically significant independent risk factors. Results are detailed in Table 3 and Figure 5.

**TABLE 3:** Multivariate analysis of factors related to rupture in type II angular pregnancy.

Parameter	β	SE	OR	95% CI	*P*-value
Abdominal pain	2.343	0.588	10.410	3.286–32.977	0.000*
Vaginal bleeding	−0.913	0.569	0.401	0.132–1.223	0.108
Ipsilateral salpingectomy	1.185	0.508	3.270	1.209–8.847	0.020*
Gestational age ≥ 7 w	−0.357	0.513	0.700	0.256–1.913	0.487

*Represents *p* < 0.05.

The results in Table 4 indicated that the median preoperative hemoglobin is 80 g/L, and 52% (13/25) of patients had a blood transfusion with hemorrhagic shock in the ruptured group, while the median preoperative hemoglobin is 127 g/L and no blood transfusion in the unruptured group. The ruptured group had clinically and statistically significantly lower hemoglobin and higher transfusion rates (*P* < 0.001).

**TABLE 4:** Significant differences in preoperative hemoglobin levels and blood transfusion rates between the ruptured and unruptured groups.

Parameter	Ruptured group (*N* = 25)	Unruptured group (*N* = 174)	*P*-value
Preoperative hemoglobin, g/L, median (IQR)	80 (71.0–96.5)	127.0 (117.3–133.0)	< 0.001*
Blood transfusion	13 (52.0)	0 (0)	< 0.001*

*Represents *p* < 0.05.

## Discussion

Finlinson et al. (13) conducted the first review on the specific signs or diagnostic criteria for angular pregnancy located at the utero-tubal junction; however, there was no detailed description of it about its categories and the factors associated with uterine rupture. Uterine rupture is a rare, life-threatening complication in 52% of blood transfusions in our study. It was reported that the incidence of uterine rupture is 5.1 per 10,000 deliveries in a scarred uterus, approximately 0.45 to 0.7 per 10,000 deliveries in an unscarred uterus, and the overall incidence of uterine rupture is approximately 0.04% to 0.09% in the general population (14–17). Our decade-long retrospective cohort analysis of 222 angular pregnancies provides critical insights into risk stratification and predictors of uterine rupture in this high-risk obstetric condition. As far as I know, it is the first to propose a novel classification system for angular pregnancy in English literature, to facilitate appropriate management and favorable prognoses. The implementation of a novel classification system (type I vs. type II) allowed for nuanced risk assessment, with type II angular pregnancy demonstrating a 12.6% rupture rate, lower than historical reports of 13.6%–28% rupture in angular pregnancies (18, 19). We reinforce the heterogeneity of angular pregnancy outcomes and emphasize the clinical utility of sub-classification in tailoring management strategies.

The multivariate analysis identified abdominal pain and prior ipsilateral salpingectomy as independent predictors of rupture in type II angular pregnancy. Notably, the absence of vaginal bleeding in ruptured cases suggests rupture may occur before significant intrauterine detachment, highlighting the limitations of relying solely on bleeding as a warning sign. These findings align with existing literature regarding the spontaneous rupture of inter-tubal pregnancies following salpingectomy but also expand its scope. Some studies, conducted on factors associated with uterine rupture for intrauterine pregnancy, revealed that salpingectomy-associated uterine rupture caused 67% fetal death (20, 21). Studies have reported angular pregnancy-associated uterine ruptures as early and as late as the 10th and 21st gestational weeks, respectively (22). In our study, the earliest and latest gestational ages reported for uterine rupture were 6th and 14.7th weeks, respectively.

Acute abdominal pain is a classical sign of uterine rupture; however, for most first and second-trimester gravidas presenting in the outpatient department, the differential diagnosis of uterine rupture may be overlooked. One study identified 61 cases of first-trimester uterine rupture with a median gestation of 11 weeks, 97% of which had abdominal pain as a presenting symptom (23). The symptom of asymmetrical pain may or may not subside during a diagnosed abnormal pregnancy, according to Jansen and associates (18). To proceed, they observed that the closer the implantation site is to the fallopian tube, the more intense the abdominal pain. Moreover, abdominal pain was one of the persistent symptoms presented by angular pregnancies in the emergency room (24). In addition, abdominal pain was identified as a hallmark of impending rupture due to tension at the uterine cornu (9), making it the most common symptom amongst the other indicators. As the gestational sac enlarges, it grows toward the tubal ostium, causing a thin myometrial layer at the uterine cornu, leading to a significantly asymmetrical and tender uterus.

Prior ipsilateral salpingectomy emerged as a significant risk factor for uterine rupture in our study, a finding not previously emphasized in other literature on angular pregnancy. Salpingectomy (partial or complete) continues to be the main treatment for ectopic pregnancies or hydrosalpinx (21). However, numerous studies have made a direct link between salpingectomy with or without cornual resection and early gestational uterine rupture (20). A previous history of salpingectomy via laparoscopy could be a risk factor for uterine rupture in pregnant women (25). We hypothesize that inflammation and fibrosis occurring in the angular region and the tissues surrounding it in patients with prior ipsilateral salpingectomy could result in reduced blood supply, decreased tissue elasticity, and restricted blood volume to the myometrium at the uterine cornual region. As the gestational sac grows and enlarges, it bulges outwards, resulting in the myometrial layer at the uterine angle becoming thinner and tense. This may reflect postsurgical anatomical changes, such as altered cornual vascularity or myometrial weakness, which could predispose to asymmetric gestational sac growth and uterine rupture. We propose that for a patient to be considered a candidate for observational management, factors such as the type presented, history of salpingectomy, and preliminary test results should be analyzed carefully.

### Strengths and limitations

To date, most of the literature on angular pregnancy consists of small case reports and review studies. Currently, there is a paucity of literature on type II angular pregnancy and the risk of rupture. The retrospective nature of our study constitutes a limitation in selection bias. Moreover, although our study was conducted by reviewing decade-long records, a type II angular pregnancy is not a common phenomenon, resulting in a relatively smaller sample size. Consequently, there is a necessity for additional large-scale prospective cohort studies to be conducted on type II angular pregnancy and the risk factors for rupture in the future.

## Conclusion

The clinical diagnosis and treatment of angular pregnancy (AP) can be challenging because of its distinct anatomic implantation. The results of APs can vary depending on the type that is present. Although type 1 AP is typically permitted for term and vaginal birth, there is a higher chance of placenta accreta at the uterine horn, necessitating the placenta’s manual removal after delivery. Pregnancy rupture is more likely in type II angular pregnancies, which are high-risk eccentric pregnancies. Thus, by classifying APs (type I and II) according to severity, physicians will be able to choose the appropriate course of treatment for their patients. Furthermore, because AP patients with a history of ipsilateral salpingectomy and abdominal pain are more likely to experience uterine rupture, we suggest timely intervention and close monitoring of such patients to avoid catastrophic outcomes.

## Data Availability

The original contributions presented in this study are included in this article/supplementary material, further inquiries can be directed to the corresponding authors.

## References

[1] Grant A Murji A Atri M. Can the presence of a surrounding endometrium differentiate eccentrically located intrauterine pregnancy from interstitial ectopic pregnancy? *J Obstet Gynaecol Can.* (2017) 39:627–34. 10.1016/j.jogc.2017.03.087 28729096

[2] Goh J Ng Z Shahul Hameed M. Hysteroscopy is a useful diagnostic and therapeutic tool for the treatment of angular pregnancy. *Gynecol Minim Invasive Ther.* (2022) 11:78–9. 10.4103/GMIT.GMIT_120_20 35310123 PMC8926045

[3] Meichen Y Jing F Lingyun Z Jianwei Z. Two cases of angular pregnancy with incomplete abortion treated with hysteroscopy: a case report and review of literature. *BMC Surg.* (2021) 21:76. 10.1186/s12893-021-01077-7 33563248 PMC7874484

[4] Kelly H. *Operative Gynecology.* New York, NY: D Appleton (1918).

[5] Yang P Shen L Ai J Zhao Y. Expectant treatment for angular pregnancy after assisted reproduction technology: a safe and patient-friendly treatment strategy. *Front Med.* (2023) 10:1234425. 10.3389/fmed.2023.1234425 37675137 PMC10477715

[6] Bollig K Schust D. Refining angular pregnancy diagnosis in the first trimester: a case series of expectant management. *Obstet Gynecol.* (2020) 135:175–84. 10.1097/AOG.0000000000003595 31809430

[7] Alves J Alves N Alencar Júnior C Feitosa F da Silva Costa F. Term angular pregnancy: successful expectant management. *J Obstet Gynaecol Res.* (2011) 37:641–4. 10.1111/j.1447-0756.2010.01405.x 21375673

[8] Chiung H Pai A Yen C. Hysteroscopic removal of a first-trimester angular pregnancy. *Gynecol Minim Invasive Ther.* (2024) 13:200–1. 10.4103/gmit.gmit_27_24 39184250 PMC11343356

[9] Chen P Liu X Fang C Zhao W. Angular pregnancy after in-vitro fertilization with timely termination to avoid uterine rupture: a case report. *Asian J Surg.* (2023) 46:2454–6. 10.1016/j.asjsur.2022.12.059 36575099

[10] Yao F Fan Y Shao L Miao P Ding H Yang M. The dilemmas in the diagnosis and management of angular pregnancy. *Taiwan J Obstet Gynecol.* (2021) 60:582–3. 10.1016/j.tjog.2021.03.040 33966757

[11] To G Kodama K Onoyama I Yahata H Kato K. Ipsilateral right angular pregnancy after a laparoscopic right salpingo-oophorectomy: a case report. *Cureus.* (2023) 15:e46171. 10.7759/cureus.46171 37905275 PMC10613323

[12] Ren C Gu X Liu X Yang Q. Expert consensus on the diagnosis and treatment of uterine angular pregnancy. *Chinese J Pract Gynecol Obstetr.* (2020) 36:329–32.

[13] Finlinson A Bollig K Schust D. Differentiating pregnancies near the uterotubal junction (angular, cornual, and interstitial): a review and recommendations. *Fertil Res Pract.* (2020) 6:8. 10.1186/s40738-020-00077-0 32391161 PMC7199330

[14] Ofir K Sheiner E Levy A Katz M Mazor M. Uterine rupture: risk factors and pregnancy outcome. *Am J Obstet Gynecol.* (2003) 189:1042–6. 10.1067/s0002-9378(03)01052-4 14586352

[15] Ronel D Wiznitzer A Sergienko R Zlotnik A Sheiner E. Trends, risk factors and pregnancy outcome in women with uterine rupture. *Arch Gynecol Obstet.* (2012) 285:317–21. 10.1007/s00404-011-1977-8 21735183

[16] Zwart J Richters J Ory F de Vries J Bloemenkamp K van Roosmalen J. Uterine rupture in The Netherlands: a nationwide population-based cohort study. *BJOG.* (2009) 116:1069–78. 10.1111/j.1471-0528.2009.02136.x 19515148

[17] Gibbins K Weber T Holmgren C Porter T Varner M Manuck T. Maternal and fetal morbidity associated with uterine rupture of the unscarred uterus. *Am J Obstet Gynecol.* (2015) 213: 382.e1-6. 10.1016/j.ajog.2015.05.048 26026917

[18] Jansen R Elliott P. Angular intrauterine pregnancy. *Obstet Gynecol.* (1981) 58:167–75. 7254728

[19] Rankin M. Angular pregnancy: a review of cases reported in the past 80 years. *Obstet Gynecol Cases Rev.* (2014) 1:3.

[20] Stanirowski P Trojanowski S Słomka A Cendrowski K Sawicki W. Spontaneous rupture of the pregnant uterus following salpingectomy: a literature review. *Gynecol Obstet Invest.* (2015) 80:73–7. 10.1159/000398795 25998257

[21] Hua Z Wu M. Spontaneous rupture of the uterus following salpingectomy: a case report and literature review. *J Int Med Res.* (2019) 47:5328–36. 10.1177/0300060519874903 31554449 PMC6833417

[22] Marfori C Kotzen M. Angular vs. interstitial pregnancy: a case report highlighting diagnostic nuances with stark management differences. *Case Rep Womens Health.* (2018) 19:e00068. 10.1016/j.crwh.2018.e00068 30094196 PMC6071364

[23] Perdue M Felder L Berghella V. First-trimester uterine rupture: a case report and systematic review of the literature. *Am J Obstet Gynecol.* (2022) 227:209–17. 10.1016/j.ajog.2022.04.035 35487324

[24] Hasanzadeh M Dadgar S Arian Y Yousefi Y. Angular ectopic pregnancy presenting as rupture of lateral wall of the uterus: late presentation in gestation week 20. *Iran J Med Sci.* (2017) 42:314–7. 28533582 PMC5429502

[25] Dong J Cao Y Ma Q Xue L Zhu W. Misdiagnosis of a twin pregnancy with double-corner uterine rupture following salpingectomy and protrusion of the amniotic sac as an adnexal cyst: a case report. *BMC Pregnancy Childbirth.* (2020) 20:71. 10.1186/s12884-020-2773-x 32013903 PMC6998286

